# Diagnostic accuracy of DNA-based SDC2 methylation test in colorectal cancer screening: a meta-analysis

**DOI:** 10.1186/s12876-022-02395-7

**Published:** 2022-06-26

**Authors:** Lixing Wang, Yu Liu, Duohan Zhang, Xiaoliang Xiong, Tingting Hao, Lili Zhong, Yinlong Zhao

**Affiliations:** 1 Department of Nuclear Medicine, The Second Hospital of Jilin University, Changchun, 130041 China; 2 Jilin Provincial Key Laboratory on Molecular and Chemical Genetic, The Second Hospital of Jilin University, Changchun, 130041 China

**Keywords:** Colorectal cancer, SDC2, Meta-analysis

## Abstract

**Background:**

A growing body of research suggests that methylated genes can be used as early diagnostic markers for cancer. Some studies on methylated Syndecan 2 (SDC2) have shown that it has a great diagnostic ability in colorectal cancer. This meta-analysis was aimed to estimate the diagnostic performance of methylated SDC2 as a potential novel biomarker to screen for the colorectal cancer.

**Methods:**

Two independent researchers conducted a comprehensive literature search to identify all relevant studies on SDC2 methylation for the diagnosis of colorectal cancer from inception to March 1, 2021. By using STATA and Revman software, the data were analyzed using a Bivariate mixed model. The quality of each study was also evaluated.

**Results:**

A total of 12 studies comprised of 1574 colorectal cancer patients and 1945 healthy people were included in our meta-analysis. Bivariate analysis showed a pooled sensitivity of 0.81 [95% confidence interval (CI) 0.74–0.86], specificity of 0.95 (95% CI 0.93–0.96), positive likelihood ratio of 15.29 (95% CI 10.83–21.60), and negative likelihood ratio of 0.21 (95% CI 0.15–0.27). The diagnostic odds ratio and the area under the summary ROC curve for diagnosing colorectal cancer were 74.42 (95% CI45.44–121.89) and 0.96 (95% CI 0.94–0.97), respectively. For adenomas, the pooled sensitivity and specificity were 0.47 (95% CI 0.34–0.61) and 0.95 (95% CI 0.92–0.97), respectively.

**Conclusions:**

Our analysis revealed that methylated SDC2 could be considered as a potential novel biomarker to screen for colorectal cancer.

**Supplementary Information:**

The online version contains supplementary material available at 10.1186/s12876-022-02395-7.

## Introduction

Colorectal cancer is the most common gastrointestinal malignancy. Some screening tools for colorectal cancer have been used to promote early detection ability, including fecal occult blood testing (FOBT) and colonoscopy [1]. As a non-invasive screening tool, FOBT has been widely utilized in the screening of colorectal cancer. However, it has a limited screening role for early colorectal cancer which has a low sensitivity for detecting stage I colorectal cancer and advanced adenoma (53%, and 27%, respectively) [2]. Colonoscopy is used as the gold standard for screening colorectal cancer because of its high sensitivity and specificity. However, colonoscopy is poorly accepted by the general public because of its need for strict bowel preparation before examination as well as unavoidable complications. Therefore, the development of more accurate screening methods to promote screening for early colorectal cancer is highly desirable.

Aberrant methylation of genes can induce silencing of tumor suppressor genes, and it is considered to be one in every of the most usual molecular alterations in colorectal cancer and other human cancers [1, 3]. Some DNA methylation biomarkers such as Septin9 (SEPT9), Secreted frizzled- related protein 2 (SFRP2), and Syndecan 2 (SDC2) from blood or stool have been considered as feasible biomarkers for early detection of colorectal cancer [4–6]. Currently, blood-based methylation of SEPT9 is the only FDA-approved biomarker for colorectal cancer screening and has been in clinical use for several years. SEPT9, however, has relatively low sensitivity (the sensitivity for CRC detection was 76.6%) for colorectal cancer detection, particularly for early cancer and advanced adenomas [7]. SDC2, on the other hand, is another biomarker found to be hypermethylated in most colorectal cancer patients [8]. SDC2 promoter region hypermethylation is a common epigenetic change during colorectal tumor development and has been successfully detected in various clinical samples such as tissue, feces, serum as well as intestinal lavage fluid. Moreover, methylated SDC2 also has a high sensitivity for the detection of early colorectal cancer and adenoma [8–12].

Some studies have assessed the diagnostic performance of SDC2 methylation in the screening of colorectal cancer, reporting different sensitivities and specificities. The purpose of this study was to explore the value of SDC2 methylation in the diagnosis of colorectal cancer by meta-analysis.

## Materials and methods

### Search strategy

To retrieve all relevant papers, a comprehensive, systematic electronic literature search of PubMed, Web of Science, Embase and OVID Medline was conducted from inception to 1^st^ March 2021. Search terms were as followed: colorectal cancer or colorectal neoplasms or colorectal carcinoma or colorectal malignant tumor and syndecan-2 or SDC2.

### Study selections

All articles were screened by two independent reviewers according to the inclusion and exclusion criteria. The inclusion criteria were: (1) The topic of the article was the study on the diagnostic accuracy of methylated SDC2 in colorectal cancer; (2) The gold standard for diagnosis in all patients with colorectal cancer was histopathology; (3) Patients did not undergo any therapy; (4) Specimens came from easily accessible sources such as blood or stool; (5) Extracted data could be used to measure true-positive (tp), false-positive (fp), false-negative (fn), and true-negative (tn) values. Articles were excluded for the following criteria: (1) Articles were not relative to the topic of our study; (2) Studies were not clinical literatures involving review articles, editorials, conference proceedings, or book chapters; (3) Data from the study were insufficient to establish 2 × 2 tables; (4) Studies were non-English literatures.

### Data extraction

For each applicable study, the following data were extracted by two independent reviewers: first author, country, year of publication, the source of specimen, methylated SDC2 detection method, cut-off, sample size, whether β-actin (ACTB) was used as reference, and number, age, gender composition of colorectal cancer and control group. Moreover, the numbers of tp, fp, fn, and tn were registered to a 2 × 2 tables. Any conflicting results between the investigators were evaluated by a third reviewer and resolved by mutual agreement.

### Quality assessment

To assess the quality of the included studies, the Quality Assessment of Diagnostic Accuracy Studies 2 (QUADAS-2) tool [13] was used by two independent reviewers, which was an evidence-based quality assessment tool for systematic reviews of diagnostic accuracy studies that included four domains: patient selection, index test, reference standard, and flow and timing. The assessment tool was operated using the Review Manager Software version 5.4.

### Statistical analysis

Heterogeneities between the studies were evaluated by measuring the I-square statistic [14]. Studies were considered homogenous if I^2^ ≤ 50%, and we performed only pooled sensitivity, specificity, positive likelihood ratio (PLR), negative likelihood ratio (NLR), and diagnostic odds ratio (DOR). If I^2^ > 50%, indicating statistically significant heterogeneity between the studies, further meta-regression and subgroup analysis were carried out to determine the possible sources of heterogeneity.

Sensitivity, specificity, accuracy, positive and negative predictive values, PLR, NLR, and DOR of methylated SDC2 in the diagnosis of colorectal cancer were obtained from the individual study, and forest plots were used to calculate and graphically display pooling of the data. The SROC curve was displayed to obtain the optimal diagnostic efficiency for methylated SDC2, and the area under the summary ROC curve (AUC) was calculated. The publication bias was presented by using the Deeks’ funnel plot asymmetry test. Meta-analysis was performed using STATA15.0 (StataCorp, College Station, TX, USA), while RevMan 5.4 (Revman, the Cochrane Collaboration) was used to evaluate the quality of the included studies. A P-value of < 0.05 was considered statistically significant.

## Results

### Literature search

**Fig. 1:**
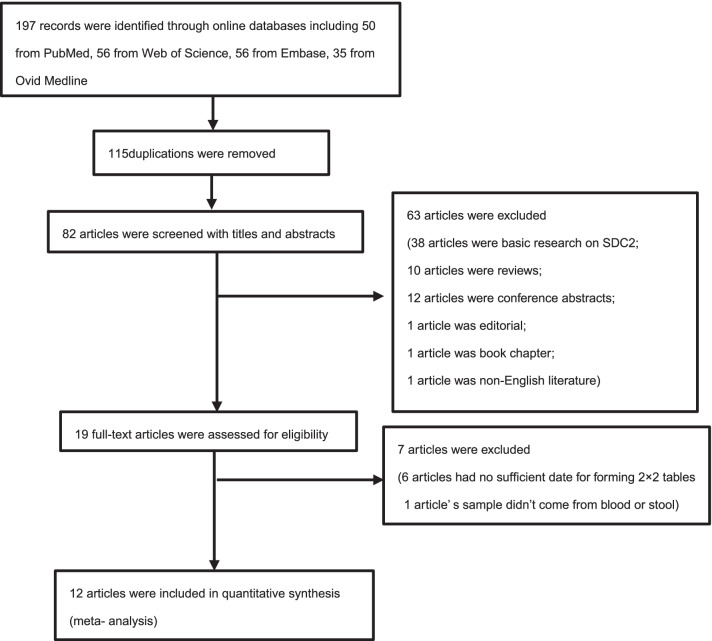
Flow diagram of the study selection process

According to the initial search of PubMed, Web of Science, Embase and OVID Medline, a total of 197 articles were identified. Of these, 115 were excluded due to repetitive publications, and 70 articles were excluded based on the inclusion/exclusion criteria: 38 were not relevant to the diagnostic accuracy of SDC2 methylation in colorectal cancer; 10 were review articles; 12 were conference abstracts; 1 was an editorial; 1 was non-English literature; 1 was book chapter; 1 use bowel lavage fluid as sample for detecting methylated SDC2; 6 were unable to be contained sufficient data to form 2 × 2 tables. Ultimately, a total of 12 studies were included in our meta-analysis (Fig. 1).

### Study characteristics

The 12 [8, 10, 11, 15–23] qualified studies involving a total of 1574 colorectal cancer patients and 1945 healthy people were included from 3 different countries: China (8/12), South Korea (3/12) and Hungary (1/12). The study type of all the included articles was retrospective. As for the source of the Methylated SDC2 detection, 5 studies measured methylated SDC2 in blood, and 7 studies in stool. There were 6 studies that used the qMSP (quantitative methylation-specific PCR) as Methylated SDC2 detection method, 3 studies used the qPCR (fluorescent quantitative PCR), 2 studies used the LTE-qMSP (linear target enrichment quantitative methylation-specific PCR), and 1 study used methylight PCR as detection method. For colorectal cancer patients and healthy controls, we extracted basic information: sample size, mean age, and gender composition. In addition, whether the ACTB was used as an internal reference and whether a cut-off was enacted were also extracted (Table 1).

**Table 1:** Major characteristics of included studies

Author	Year	Country	Sample source	Methylated SDC2 detection methods	Cut-off	colorectal cancer case			Control			Sample size	ACTB for reference
						N	Age (y)	M/F	N	Age (y)	M/F		
TaeJeong Oh	2013	South Korea	Blood	qMSP	PMR > 0.936	131	58.4 (33–84)	69/62	125	51.0 (40–61)	64/61	256	Yes
Barbara Kinga Barták	2017	Hungary	Blood	MethyLight PCR	NR	37	NR	NR	47	NR	NR	121	No
Tae Jeong Oh	2017	South Korea	Stool	LTE-qMSP	C_T_ < 40	50	61.9 (41–84)	30/20	22	58.8 (36–77)	12/10	72	No
Feng Niu	2017	China	Stool	qMSP	NR	196	61.0 (43–79)	121/75	179	61.0 (45–76)	70/109	375	Yes
Minghao Sun	2019	China	Stool	qPCR	C_T_ < 42	105	NR	NR	108	NR	NR	213	Yes
Guodong Zhao	2019	China	Blood	qMSP	Cp < 50	117	61.8 (25–89)	64/53	166	36.6 (21–69)	87/79	283	Yes
Ying Chen	2019	China	Blood	qMSP	Cp < 50	111	61.0 (25–89)	NR	114	33.2 (19–60)	NR	225	Yes
Yoon Dae Han	2019	South Korea	Stool	LTE-qMSP	C_T_ < 40	245	NR	133/122	245	NR	114/131	490	No
Guodong Zhao **I**	2020	China	Blood	qMSP	Cp < 44	122	61.8 (30–88)	64/58	91	39.3 (23–69)	56/35	213	Yes
Guodong Zhao **II**	2020	China	Stool	qPCR	C_T_ < 50	39	59.0 (27–83)	21/18	59	47.9 (24–83)	31/28	98	Yes
Jianping Wang	2020	China	Stool	qMSP	C_T_ < 38	359	NR	234/125	713	NR	NR	1072	Yes
Wei-Chih Su	2021	China	Stool	qPCR	C_T_ < 39	62	NR	33/29	76	NR	37/39	138	No

*SDC2* Syndecan-2, *ACTB* β-actin, qMSP quantitative methylation-specific PCR, *LTE* linear target enrichment, *qPCR* fluorescent quantitative PCR, *PMR* percentage of methylated reference

### Risk of bias and quality assessment

Publication bias was analyzed by using the Deek’s funnel chart. The funnel chart was well-proportioned with a P-value of 0.15 suggesting that there was no apparent publication bias between the included studies (see Additional file1: Fig. S1). The analysis using the QUADAS-2 tool showed a low risk of bias and moderate to a high quality of the included studies (see Additional file2: Fig. S2).

### Diagnostic effect

**Fig. 2:**
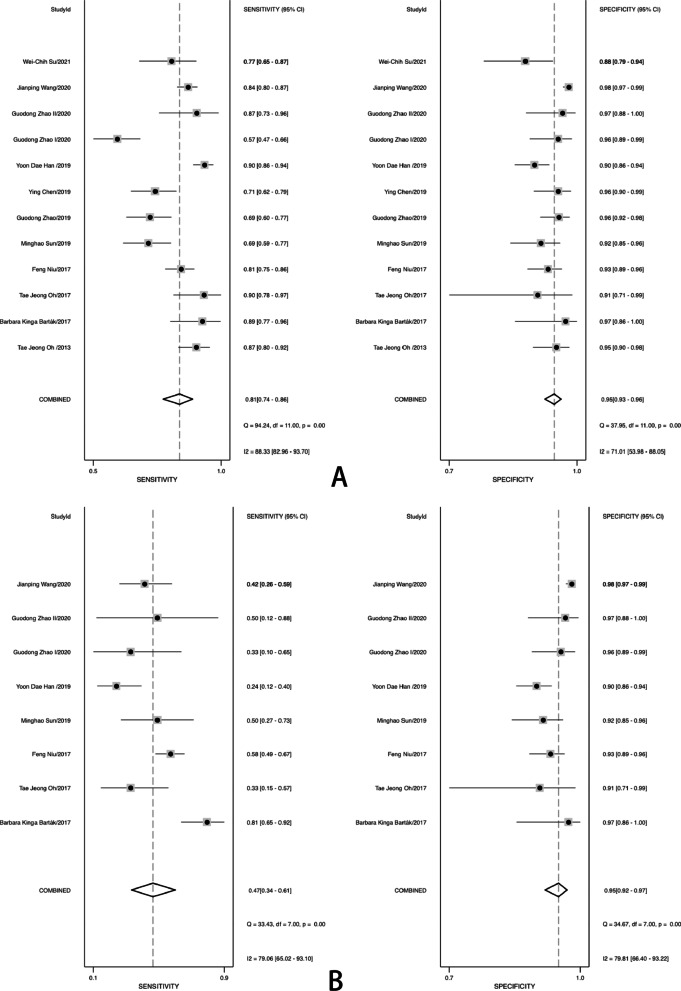

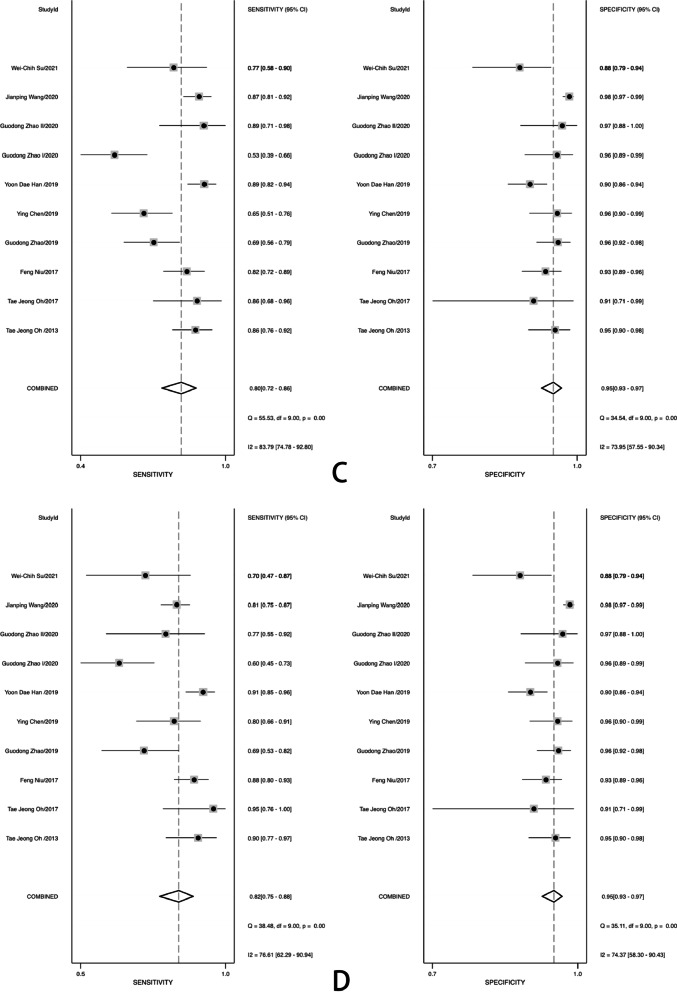
Forest plots of pooled sensitivity and specificity. **A** All stage CRC; **B**, adenoma; **C** stage I and II CRC; **D** stage III and IV CRC

**Fig. 3:**
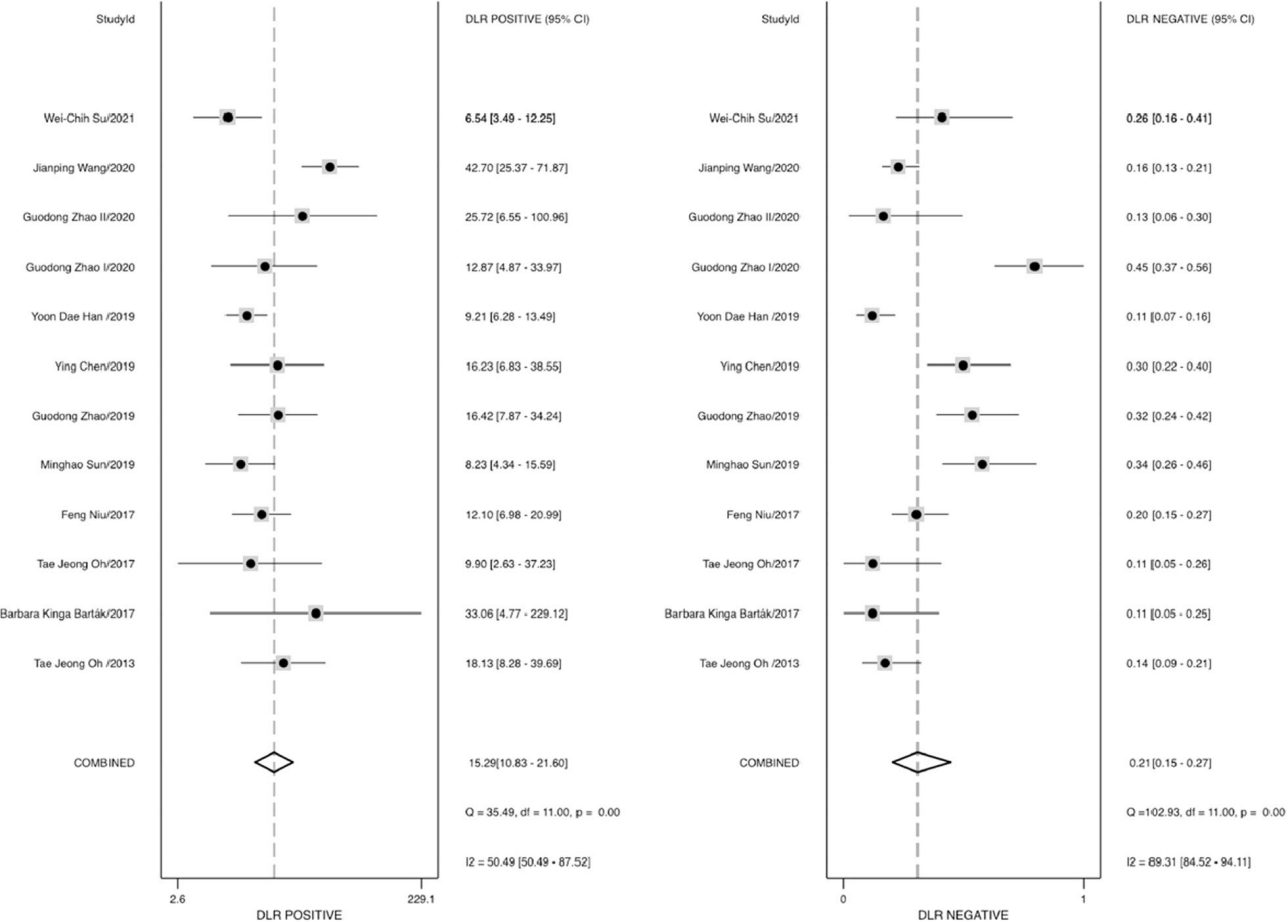
Forest plots of pooled positive likelihood radio and negative likelihood ratio

**Fig. 4:**
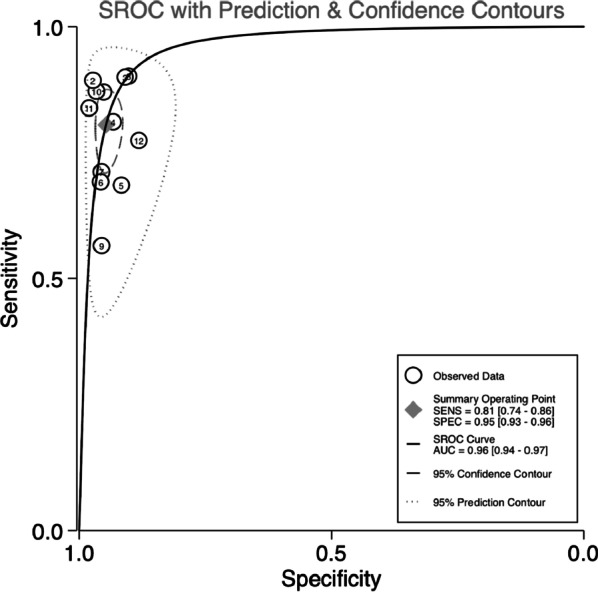
SROC curve of methylated SDC2 for the diagnosis of colorectal cancer

The pool sensitivity and specificity of methylated SDC2 for diagnosing colorectal cancer of all stage were 0.81 (95% CI 0.74–0.86) and 0.95 (95% CI 0.93–0.96), respectively (Fig. 2A), while the pooled PLR and NLR (Fig. 3) were 15.29 (95% CI 10.83–21.60) and 0.21 (95% CI 0.15–0.27), respectively. Furthermore, the pooled DOR (see Additional file3: Fig. S3) was 74.42 (95% CI45.44–121.89) and AUC (Fig. 4) was 0.96 (95% CI 0.94–0.97). In addition, for stage I, II (10 studies containing 729 patients) and stage III, IV (10 studies containing 685 patients) of colorectal cancer, the pooled sensitivity were 0.80 (95% CI 0.72–0.86) and 0.82 (95% CI 0.75–0.88), respectively (Fig. 2C, D). For adenoma (8 studies containing 297 adenoma patients), the pooled sensitivity was 0.47 (95% CI 0.34–0.61) (Fig. 2B).

### Meta-regression and subgroup analysis

The sensitivity and specificity of I^2^ were 88.33% and 71.01%, respectively (Fig. 2A). Therefore, there was significant heterogeneity among studies. To investigate potential sources of heterogeneity existing in our study, univariate meta-regression and subgroup analyses were performed. Country, sample source (blood, stool), sample size, cut-off, ACTB for reference, and population descriptions were included in the meta-regression analysis of sensitivity and specificity (see Additional file4: Fig. S4). The forest plots of univariate meta-regression indicated that country, sample size, and population descriptions could be the sources of the heterogeneity in sensitivity, whereas country, sample source, sample size, cut-off, ACTB for reference and population descriptions could be the sources of heterogeneity in specificity.

Factors including country, sample source, sample size, ACTB for reference, and population descriptions were included in the subgroup analysis (Table 2). The sensitivity in China was lower than other countries containing South Korea and Hungary (0.77, 95% CI 0.70–0.82: and 0.89, 95% CI 0.86–0.93, respectively), however, the specificity in China was better than that in other countries (0.95, 95% CI 0.93–0.97; and 0.93, 95% CI 0.88–0.96, respectively). With regards to the source of sample, there was no significant difference in sensitivity (0.82, 95% CI 0.58–0.94; and 0.83, 95% CI 0.77–0.88, respectively) or specificity (0.95, 95% CI 0.91–0.97; and 0.94, 95% CI 0.90–0.96, respectively) between samples deprived from blood and stool. For population descriptions, detailed populations descriptions performed better in terms of sensitivity (0.82, 95% CI 0.72–0.88; and 0.78, 95% CI 0.70–0.83, respectively) than non-detailed descriptions. The specificity (0.96, 95% CI 0.94–0.97; and 0.90, 95%CI 0.87–0.93, respectively) of using the ACTB as an internal reference was better than that of not using the ACTB, but the sensitivity (0.77, 95% CI 0.69–0.83; and 0.88, 95% CI 0.82–0.92, respectively) is less than that of not using the ACTB as an internal reference. Both the sensitivity and specificity in sample size > 250 (0.83, 95% CI 0.77–0.88; and 0.95, 95% CI 0.92–0.97, respectively) were higher than that in sample size < 250 (0.82, 95% CI 0.67–0.91; and 0.92, 95% CI 0.89–0.95, respectively).

**Table 2:** Subgroup analysis of diagnostic effect

Subgroup	No. studies	Sample size	Sensitivity	Specificity	Diagnostic odds ratio
			Value	Value	Value
*Country*					
China	8	2689	0.77 (0.70–0.82)	0.95 (0.93–0.97)	63 (35–115)
Other countries	4	902	0.89 (0.86–0.93)	0.93 (0.88–0.96)	107 (61–189)
*Sample source*					
Blood	5	1061	0.82 (0.58–0.94)	0.95 (0.91–0.97)	82 (31–217)
Stool	7	2458	0.83 (0.77–0.88)	0.94 (0.90–0.96)	77 (39–149)
*Population description*					
Detailed	7	1787	0.82 (0.72–0.88)	0.94 (0.91–0.96)	68 (45–105)
Undetailed	5	1732	0.78 (0.70–0.83)	0.96 (0.91–0.98)	74 (26–207)
*ACTB for reference*					
Yes	8	2735	0.77 (0.69–0.83)	0.96 (0.94–0.97)	73 (39–137)
No	4	784	0.88 (0.82–0.92)	0.90 (0.87–0.93)	67 (36–127)
*Sample size*					
> 250	5	2476	0.83 (0.77–0.88)	0.95 (0.92–0.97)	102 (59–178)
< 250	7	1043	0.82 (0.67–0.91)	0.92 (0.89–0.95)	55 (27–122)

*ACTB* β-actin. Other countries: South Korea and Hungary

## Discussion

Colorectal cancer is one of the most prevalent malignancies worldwide and one of the leading causes of cancer-related death. According to epidemiological studies in developed countries, long-term screening and early detection of colorectal cancer have played an important role in reducing morbidity and mortality [24]. In the United States, the mortality rate of colorectal cancer has decreased by more than 50% in the past 40, which is mainly due to increased screening of the population [24, 25]. Some screening tools have been developed, but these examination methods are not all perfect. FOBT is the most extensively used screening method. Although its application can reduce the mortality, its sensitivity is limited in detecting early colorectal cancer, especially for advanced adenoma [2]. Because of its high sensitivity and specificity as the gold standard for the early diagnosis of colorectal cancer, colorectal colonoscopy can be used to detect early colorectal cancer and can treat polyps or adenomas at the same time of examination. [12]. However, screening colonoscopy is not well accepted by the public due to strict bowel preparation before the examination, invasiveness during the examination as well as unavoidable complications [26]. Therefore, the development of a non-invasive, convenient, and accurate molecular diagnostic technique that can sensitively and specifically detect tumor characteristics of colorectal cancer (e.g., DNA methylation) can improve screening rates in the population and reduce mortality.

Abnormal DNA methylation of specific genes may be an early event in the process of tumorigenesis and could be used as a biological marker to diagnose cancer early. [27]. It has been reported that biomarkers from feces or blood can be detected very early by DNA methylation of multiple promoters, which can also be used as a tool for monitoring colorectal cancer [28]. Although as the only blood-based biomarker approved by the FDA for colorectal cancer screening, the sensitivity of methylated SPET9 for detecting stage I colorectal cancer was as low as 35.0%, and the sensitivity for detecting adenoma ranged from 9.8 to 21.6%, which indicates that methylated SEPT9 is not excellent for screening of early colorectal cancer and adenoma [7, 29, 30].

The Syndecan2 protein is a transmembrane heparan sulfate proteoglycan with a key role in the regulation of cell proliferation, migration, and cell–matrix interactions through its interaction with extracellular matrix proteins [31]. Some studies have shown that the hypermethylation of SDC2 is found in some malignant tumor tissues, such as the hypermethylation of SDC2 can be detected in gastric cancer tissues, and it can also be seen in head and neck squamous cell carcinoma. Moreover, downregulation of SDC2 has also been found to be associated with poor prognosis in esophageal scale-cell carcinoma [32–34]. Recently, hypermethylation of SDC2 has also been found in the feces or blood samples of most colorectal cancer patients, indicating its potential as a non-invasive molecular diagnostic biomarker for early detection. And studies have shown that methylated SDC2 also has a high sensitivity for the detection of early colorectal cancer and advanced adenoma [8, 35].

Although previous meta-analyses [36] and systematic reviews [37] have analyzed and evaluated SDC2 methylation derived from feces and blood, respectively, for colorectal cancer screening, they included a small number of articles and lacked specific study analysis of SDC2 methylation. Our study was the first meta-analysis to investigate the performance of SDC2 methylation for colorectal cancer screening.

From data extracted from the included studies, we assessed the diagnostic accuracy of methylation SDC2 as a colorectal cancer screening tool by calculating sensitivity, specificity, PLR, NLR and DOR. Our analysis showed that the combined sensitivity and specificity of SDC2 methylation for colorectal cancer diagnosis was 0.81 and 0.95, respectively. In addition, the diagnostic performance was assessed by calculating the SROC curve, which showed an AUC of 0.96, indicating superb diagnostic capability of SDC2 methylation. As an indicator of the accuracy of the test, our analysis showed a DOR of 74.42, revealing good differentiating ability. Finally, the likelihood ratio and post-test probability demonstrated the risk of colorectal cancer in the event of a positive or negative test result. Our results showed a PLR of 15.29 and an NLR of 0.21, indicating that colorectal cancer patients were 15 times more likely to test positive than healthy individuals. These results suggested that methylation of SDC2 represented a promising method for the diagnosis of colorectal cancer.

Moreover, we analyzed the diagnostic ability of SDC2 methylation in different stages of colorectal cancer, and the results showed that methylated SDC2 showed extraordinary ability in diagnosing stage I and II colorectal cancer (sensitivity: 0.80), and there was no significant difference in diagnostic efficacy compared with stage III and IV colorectal cancer (sensitivity: 0.82). SDC2 also evaluated the screening ability of adenomas, and a total of 297 patients with adenomas (adenoma diameter including > 1 cm and < 1 cm) from 8 articles were synthesized for data. The results showed that methylated SDC2 also had some screening ability for adenoma. Overall, methylated SDC2 showed better efficacy than methylated SEPT9 for screening both early colorectal cancer and adenoma. Multitarget fecal DNA (MT-sDNA) was also a non-invasive colorectal cancer screening method and has shown good screening ability (Sensitivity 92%, specificity 87%) [38]. SDC2 methylation showed slightly lower sensitivity and higher specificity in detecting CRC compared to MT-sDNA.

Since the heterogeneity of our study was derived from non-threshold effects, we performed meta-regression and subgroup analysis to find the source of heterogeneity. The results showed that populations from different countries might be the source of heterogeneity, and that SDC2 methylation had poorer screening efficacy for colorectal cancer in populations derived from China than in other countries. The size of the sample included in the study might also be a source of heterogeneity, and we found that studies with sample size greater than 250 showed higher diagnostic sensitivity and specificity. Whether there was a detailed description of the population might also be a source of heterogeneity. We defined the detailed population description as follows: The included colorectal cancer patients and healthy controls provided mean age, gender composition, and sample size. The study in which we found a detailed population description showed the higher sensitivity of SDC2 methylation for colorectal cancer diagnosis. The reason for this result might be that the incidence of cancer varied in different age groups and genders, and whether the age and gender composition of patients and controls were considered in detail in the design of the experiment might have an impact on the final diagnostic efficacy of the study.

Our subgroup analysis also showed that whether ACTB was used as a reference also affected the diagnostic efficacy of SDC2 methylation for colorectal cancer. ACTB was used as an internal control gene for effective sample collection and processing to standardize DNA input in order to confirm the quality and quantity of bisulfite modified serum DNA and avoid false negatives. The results showed that the sensitivity of using ACTB as the internal reference was lower than that without ACTB as the reference, while the specificity was higher than that without ACTB as the reference. Meta-regression showed that the source of samples might be a specific source of heterogeneity. However, the results of subgroup analysis showed that SDC2 methylation in samples derived from blood and feces did not differ much in colorectal cancer screening ability.

Compared with traditional colorectal cancer screening methods, methylated SDC2 demonstrated high sensitivity and specificity, especially for early colorectal cancer and adenoma. However, our study had some shortcomings. First, most of the studies we included were case–control or cross-sectional nature, and more prospective studies were lacking to investigate SDC2 methylation. Second, most of the current poor studies were limited to Asian populations, and to comprehensively assess their power, more clinical trials in different ethnic populations were needed to provide more comprehensive data. Finally, as adenoma is a precancerous lesion of colorectal cancer, but there was still a lack of research on methylated SDC2 in the diagnosis of adenoma. Therefore, more clinical studies targeting adenomas were needed to provide more comprehensive information.

## Conclusion

The pooled results of our meta-analysis have confirmed the difference in the SDC2 methylation between patients with tumors of colorectal and healthy individuals, which sheds light on SDC2 methylation as a promising novel screening biomarker for early detection of colorectal cancer.

## Supplementary Information

**Additional file 1**. **Fig. S1**: Deek funnel plot for the assessment of the publication bias.

**Additional file 2**. **Fig. S2**: A risk of bias and applicability concerns graph, B risk of bias and applicability concerns summary.

**Additional file 3**. **Fig. S3**: Forest plots of DOR of methylated SDC2 for the diagnosis of colorectal cancer.

**Additional file 4**. **Fig. S4**: Forest plot of meta-regression and subgroup analyses of sensitivity and specificity methylated SDC2 in screening colorectal cancer.

## Data Availability

All data generated or analyzed during this study are included in this published article. If anyone would like to request data, please contact the corresponding author.

## Abbreviations

95% CI: 95% Confidence interval
FOBT: Fecal occult blood testing
SEPT9: Septin9
SFRP2: Secreted frizzled- related protein 2
SDC2: Syndecan 2
tp: True-positive
fp: False-positive
fn: False-negative
tn: True-negative
ACTB: β-Actin
PLR: Positive likelihood ratio
NLR: Negative likelihood ratio
DOR: Diagnostic odds ratio
AUC: The area under the summary ROC curve
