# Supplementary figures and images for: Diagnostic accuracy of DNA-based SDC2 methylation test in colorectal cancer screening: a meta-analysis
Source: BMC Gastroenterol. 2022 Jun 26;22:314. doi: 10.1186/s12876-022-02395-7 (PMC9235166; doi:10.1186/s12876-022-02395-7)

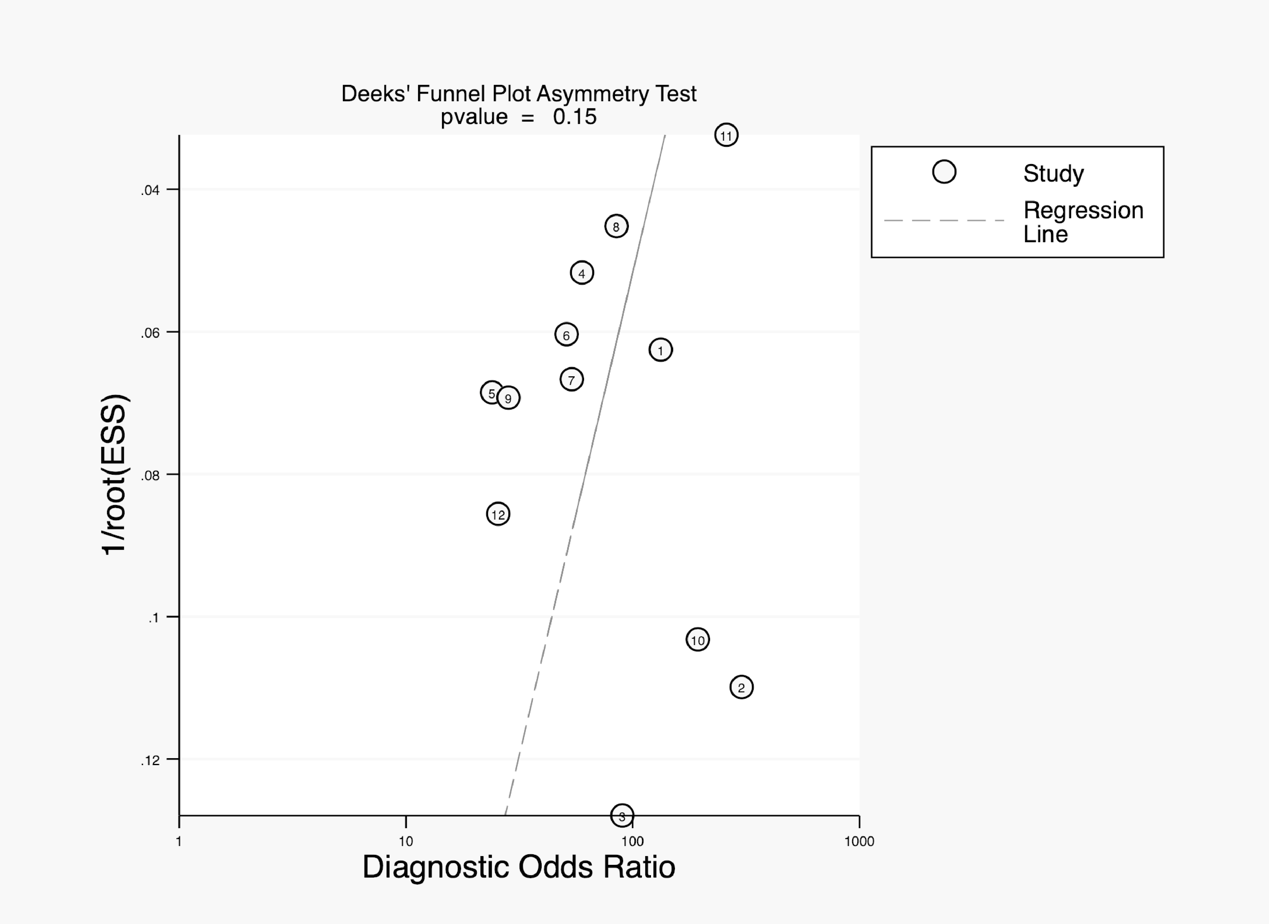


Supplemental Figure 1. Deek funnel plot for the assessment of the publication bias.

Supplement: Supplementary file 1 — Additional file 1. Fig. S1: Deek funnel plot for the assessment of the publication bias. [file 12876_2022_2395_MOESM1_ESM.docx]
