# Supplementary material for: Diagnostic accuracy of DNA-based SDC2 methylation test in colorectal cancer screening: a meta-analysis
Source: BMC Gastroenterol. 2022 Jun 26;22:314. doi: 10.1186/s12876-022-02395-7 (PMC9235166; doi:10.1186/s12876-022-02395-7)
Supplement: Supplementary file 2 — Additional file 2. Fig. S2: A risk of bias and applicability concerns graph, B risk of bias and applicability concerns summary. [file 12876_2022_2395_MOESM2_ESM.docx]

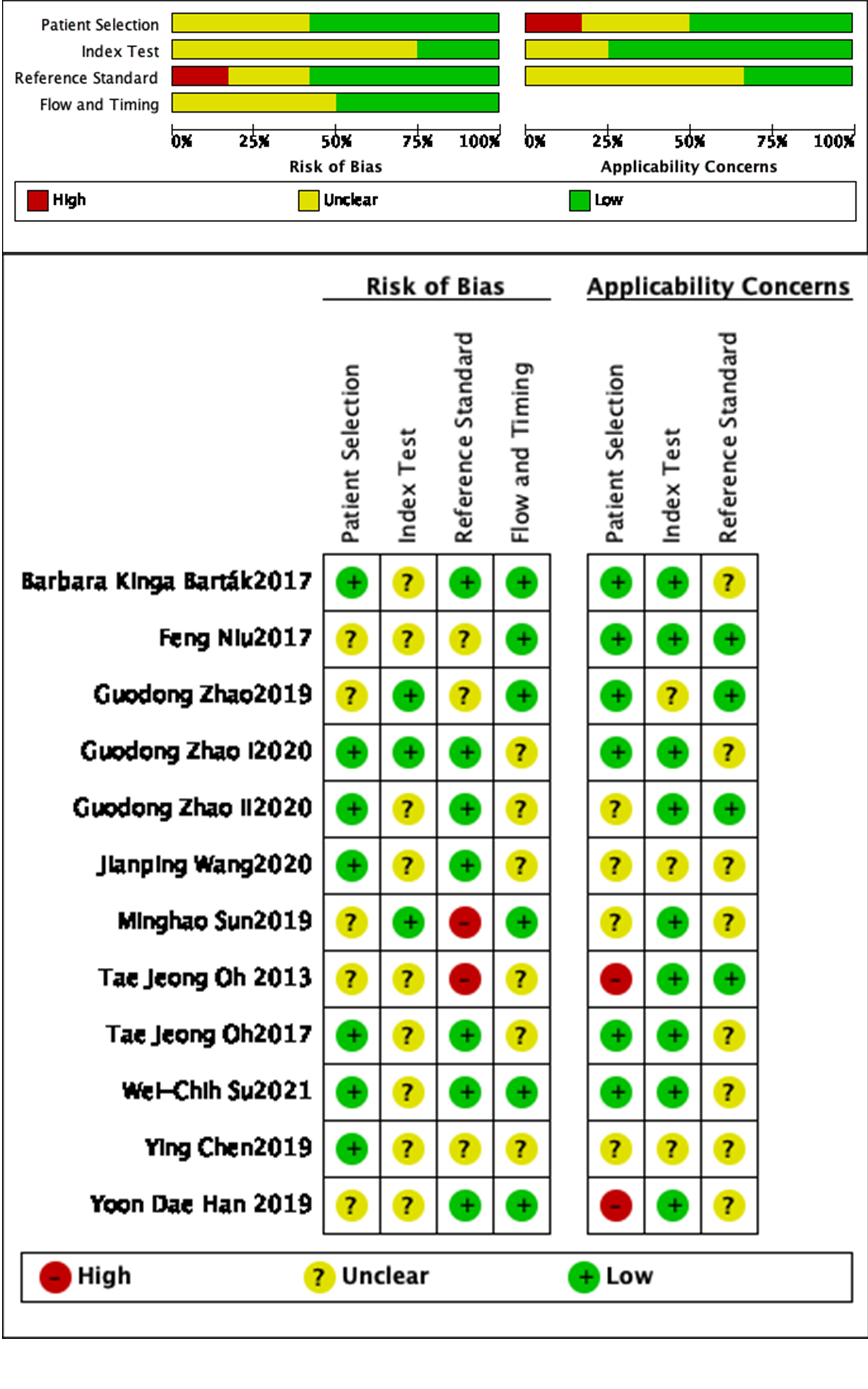


Supplemental Figure 2. A risk of bias and applicability concerns graph, B risk of bias and applicability concerns summary.
