# Supplementary material for: Diagnostic accuracy of DNA-based SDC2 methylation test in colorectal cancer screening: a meta-analysis
Source: BMC Gastroenterol. 2022 Jun 26;22:314. doi: 10.1186/s12876-022-02395-7 (PMC9235166; doi:10.1186/s12876-022-02395-7)
Supplement: Supplementary file 3 — Additional file 3. Fig. S3: Forest plots of DOR of methylated SDC2 for the diagnosis of colorectal cancer. [file 12876_2022_2395_MOESM3_ESM.docx]

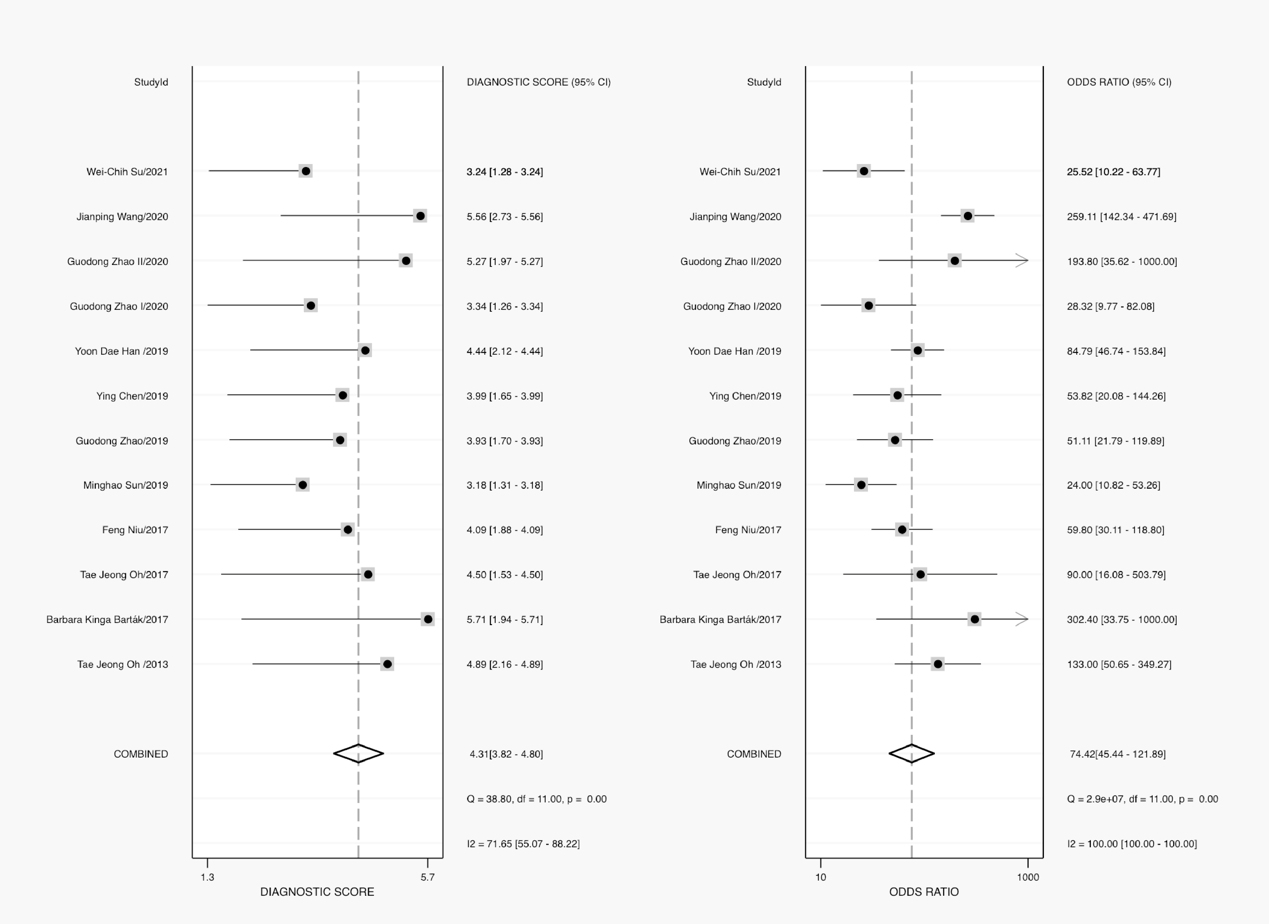


Supplemental Figure 3. Forest plots of DOR of methylated SDC2 for the diagnosis of colorectal cancer.
