# Supplementary material for: Diagnostic accuracy of DNA-based SDC2 methylation test in colorectal cancer screening: a meta-analysis
Source: BMC Gastroenterol. 2022 Jun 26;22:314. doi: 10.1186/s12876-022-02395-7 (PMC9235166; doi:10.1186/s12876-022-02395-7)
Supplement: Supplementary file 4 — Additional file 4. Fig. S4: Forest plot of meta-regression and subgroup analyses of sensitivity and specificity methylated SDC2 in screening colorectal cancer. [file 12876_2022_2395_MOESM4_ESM.docx]

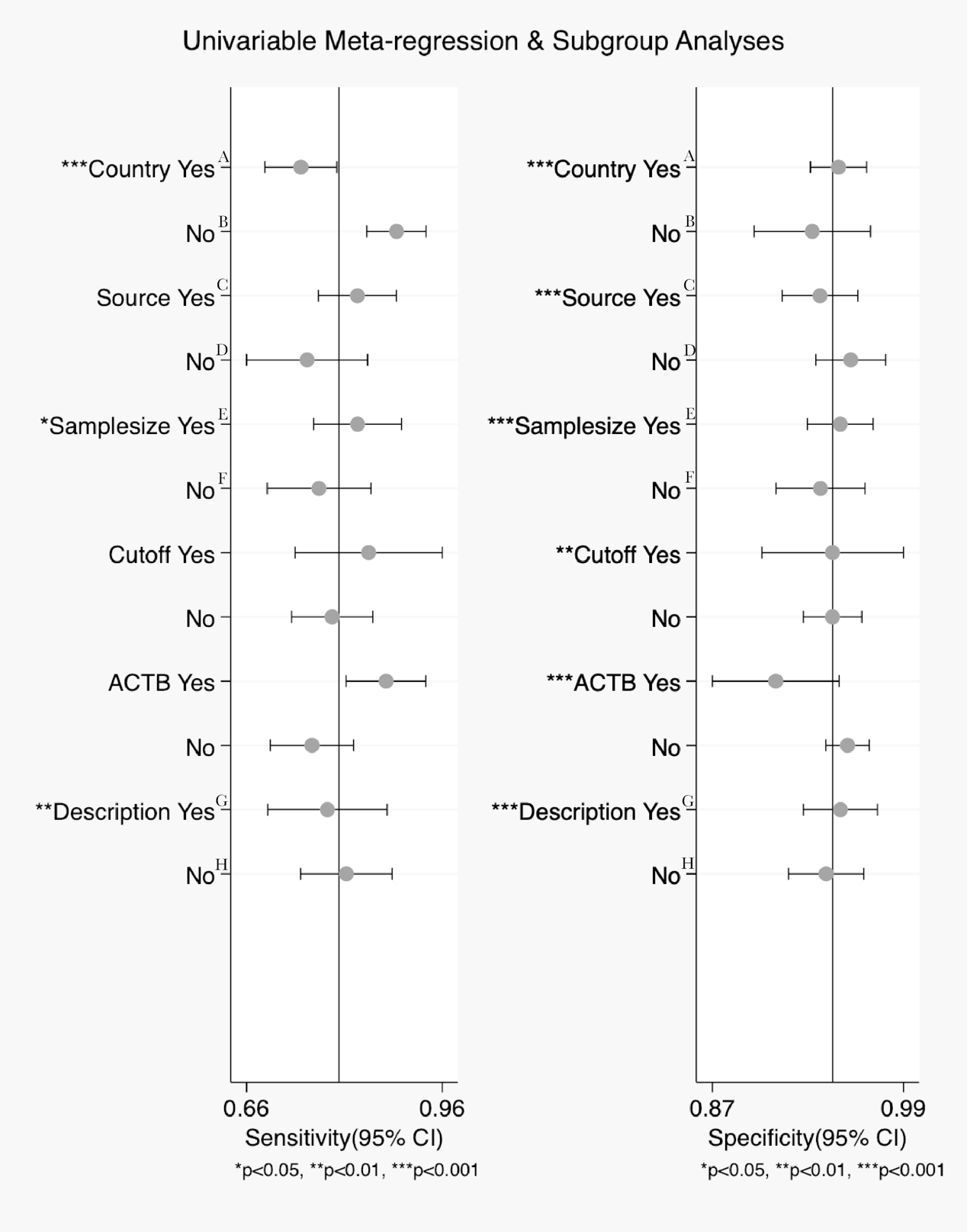


Supplemental Figure 4. Forest plot of meta-regression and subgroup analyses of sensitivity and specificity methylated SDC2 in screening colorectal cancer.

Abbreviations: (A)China; (B) Other countries; (C)stool; (D)blood; (E)sample size>250; (F)sample size<250; (G)undetailed; (H)detailed.
